# Multi-modal data integration reveals functionally credible predictive biomarkers in ovarian cancer

**DOI:** 10.1186/s13073-026-01706-x

**Published:** 2026-07-03

**Authors:** Taru A. Muranen, Andreas Hainari, Daria Afenteva, Wojciech Senkowski, Jaana Oikkonen, Johann Dreo, Kyriaki Driva, Susanna Holmström, Veli-Matti Isoviita, Alexandra Lahtinen, Kari Lavikka, Yilin Li, Marta Lovino, Ilari Maarala, Giovanni Marchi, Francesca Miccolis, Giulia Micoli, Matthieu Najm, Ann-Christin Ostwaldt, Pablo Rodriguez-Mier, Jenni Söderlund, Sakari Hietanen, Anni Virtanen, Julio Saez-Rodriguez, Elisa Ficarra, Benno Schwikowski, Erika Alanne, Krister Wennerberg, Sampsa Hautaniemi, Johanna Hynninen

**Affiliations:** 1 Research Program in Systems Oncology, Research Programs Unit, Faculty of Medicine, University of Helsinki, Helsinki, Finland; 2 Biotech Research & Innovation Centre, University of Copenhagen, Copenhagen, Denmark; 3 Computational Systems Biomedicine Lab, Institut Pasteur, Universite Paris Cité, Paris, France; 4 Department of Obstetrics and Gynecology, University of Turku and Turku University Hospital, Turku, Finland; 5 Department of Life Sciences, University of Modena and Reggio Emilia, Modena, Italy; 6 Department of Engineering, Enzo Ferrari, University of Modena and Reggio Emilia, Modena, Italy; 7 Faculty of Medicine and Heidelberg University Hospital, Institute for Computational Biomedicine, Heidelberg University, Heidelberg, Germany; 8 Department of Pathology, University of Helsinki and HUS Diagnostic Center, Helsinki, Finland; 9 European Bioinformatics Institute (EMBL-EBI), Hinxton, Cambridgeshire UK; 10 Department of Oncology, Turku University Hospital and Western Finland Cancer Center, Turku, Finland

**Keywords:** Precision oncology, Targeted therapy, Mutation, Biomarker, High-grade serous carcinoma, Ovarian cancer, Multi-omics

## Abstract

**Background:**

Precision oncology aims to tailor treatment according to tumor-specific molecular alterations, but the success of aberration-guided therapies has been limited in clinical trials. Here, we develop an integrated whole-genome and transcriptome workflow to systematically distinguish functionally credible, predictive driver aberrations from non-functional alterations across all classes of genomic events.

**Methods:**

We applied the integrated omics workflow to 335 patients with ovarian high-grade serous carcinoma (HGSC) enrolled in the observational DECIDER study. Tumor samples were collected from multiple cancer sites as part of the standard cancer care. DNA and RNA were extracted together from snap-frozen tumor samples and sent to whole-genome and transcriptome sequencing. Sequencing data were processed with the Anduril 2 pipeline for detection and validation of short somatic changes and with the HMW toolkit and the nf-core/rnafusion pipeline for assessment of structural changes. Aberration-specific drug sensitivity was tested in patient-derived organoids with a drug screen combining targeted agents and chemotherapy.

**Results:**

Using an agnostic integrated omics analysis, we identified clinically relevant ESCAT Tier II–III alterations in more than 40% of the patients, even though 58% of all nominally pathogenic variants proved to be false positives. Credible aberrations were predominantly clonal, detected across anatomical sites, and preserved from diagnosis to relapse, indicating early establishment during tumor evolution. The most recurrent actionable event was NF1 deficiency, which was associated with a robust transcriptional footprint and marked sensitivity to KRAS- and MEK-inhibition in patient-derived organoids. Notably, integrated DNA-RNA analysis enabled discrimination of treatment-guiding aberrations from false-positive findings that would otherwise misinform treatment selection and confound clinical trial outcomes.

**Conclusions:**

Our findings provide a strategy for more reliable biomarker detection in precision oncology, inform biomarker-guided clinical trial design, and reveal unexploited therapeutic vulnerabilities in HGSC.

**Supplementary Information:**

The online version contains supplementary material available at https://doi.org/10.1186/s13073-026-01706-x.

## Background

Precision oncology promises improved cancer management by leveraging individual, cancer-specific driver mutations to guide targeted therapies [1]. Recently, the precision oncology paradigm has been evaluated in large pan-cancer trials, including NCI-MATCH [2], DRUP [3], and SCRUM-MONSTAR [4], where thousands of cancer patients have received targeted therapies guided by genomic biomarkers. These studies show that while actionable aberrations are detected in about a third of patients, clinical benefits vary largely between cancer types, with overall response rates reported between 10 and 33% and survival benefit of only a few months.

Most targeted therapies act on specific genomic alterations, which are assumed to either be essential to continuous tumor cell proliferation via oncogene addiction or cause detrimental cellular deficiencies [5, 6]. However, the identification of such central events is complicated by inert passenger mutations or co-driver aberrations with synergistic oncogenic effects [2]. Consequently, the precision oncology trials are challenged by false negative cases, where an existing driver event remains undetected, and by false positives, where nominally actionable aberrations lack functional effect in tumor cells. Indeed, several studies have suggested that combining whole-genome and transcriptome sequencing would enhance the detection of actionable biomarker aberrations [7–11], although their focus remains on expanding the number of potential actionable alterations rather than improving precision by systematically assessing the functional impact of genomic alterations.

Here, we report a precision oncology workflow that combines whole-genome and transcriptome sequencing data to comprehensively identify targetable molecular alterations and to distinguish credible driver events from redundant passenger aberrations. We applied the workflow to characterize the actionable landscape of prospectively collected patients with ovarian high-grade serous carcinoma (HGSC), which is a copy number-driven cancer with nearly universal TP53 dysfunction [12, 13], and five-year survival rate below 40% [14]. Current actionable molecular biomarkers in HGSC are limited to *BRCA1/2* gene mutation and homologous recombination deficiency (HRD) [15]. However, upon development of resistance to the standard platinum-based chemotherapy, few efficient treatment options remain.

Our findings, based on a real-world cohort of HGSC cases, show that tumors of about 40% of patients with HGSC harbor unexploited targetable aberrations and that incorporating RNA-level data considerably improves the detection of credible aberrations. Furthermore, our pre-clinical research in patient-derived organoids supports the use of KRAS or MEK inhibitors for patients with NF1-deficient tumors.

## Methods

### Patients and samples

The analysis was based on real-world data on patients with ovarian high-grade serous carcinoma enrolled in the prospective, observational, longitudinal DECIDER study between October 2009 and September 2024. Patients were treated at Turku University Hospital and stratified to primary debulking surgery (PDS) followed by adjuvant carboplatin and paclitaxel chemotherapy or to neoadjuvant chemotherapy (NACT) followed by interval debulking surgery and adjuvant chemotherapy. Follow-up was centralized to Turku University Hospital for five years after diagnosis as part of routine cancer care.

**Fig. 1:**
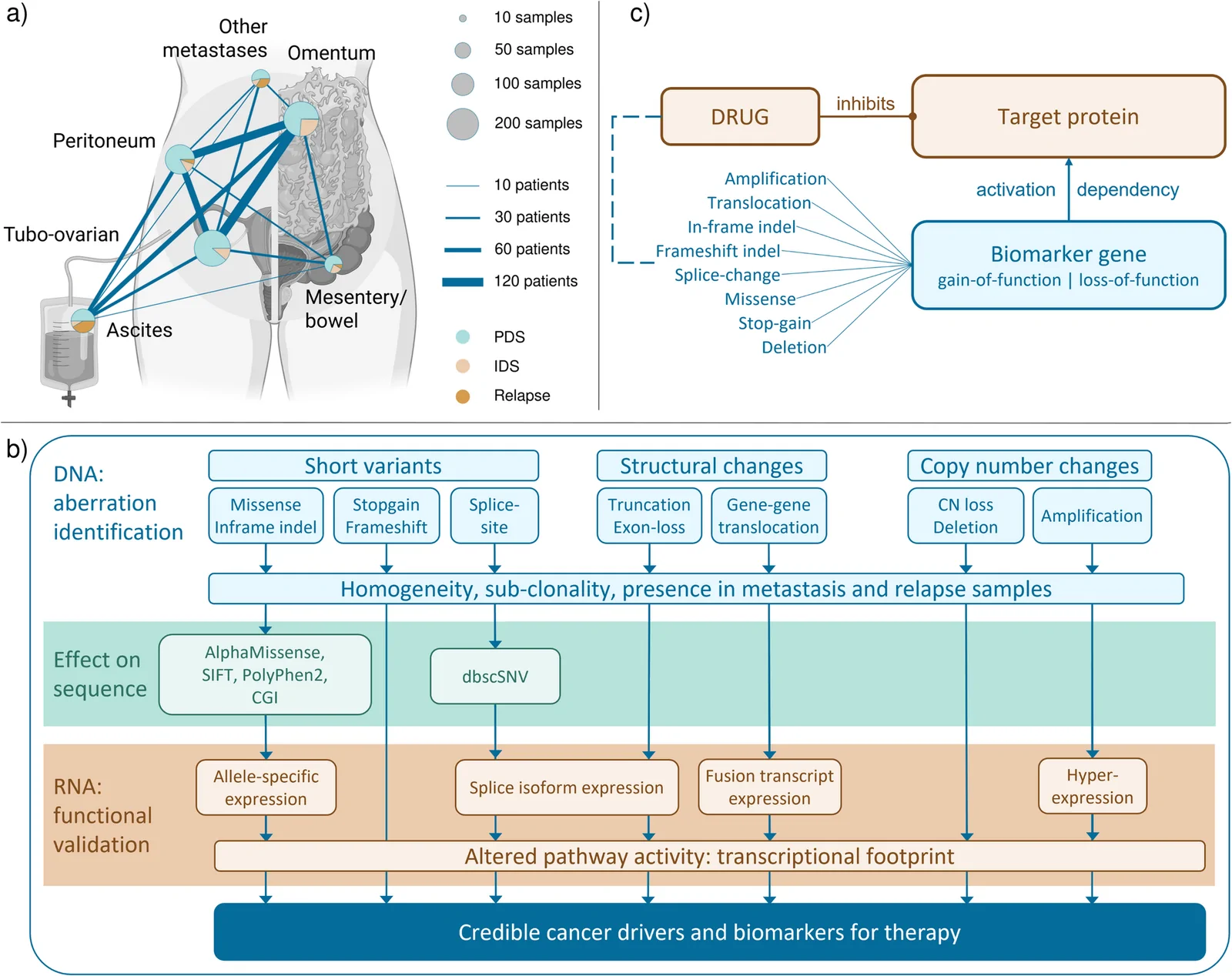
Overview of the multi-omics workflow and its application to HGSC samples. **a** Tumor samples from primary and metastatic sites and ascites were collected from patients with HGSC for whole-genome and transcriptome sequencing. Pie charts describe the number of samples from each tumor site category and the proportion of samples from primary debulking surgery (PDS), interval debulking surgery (IDS) and palliative relapse operations. Edges between the pie charts describe the frequency of the two sites having been analyzed from the same patient. **b** Schematic representation of the multi-omics workflow for systematic evaluation of somatic genomic aberrations. **c** Logical and functional relationships between drugs, their target proteins, associated biomarker genes, and the different types of aberrations (solid lines) enabled connecting all somatic aberrations to targeted cancer drugs (dashed line)

Solid tumor, ascites, or pleural fluid samples were collected during the standard care. Nucleic acids were extracted from snap-frozen tumor samples and sequenced as previously described [16–18] and summarized in the Supplementary methods (Additional file 1). Quality-controlled tumor whole-genome and transcriptome sequencing data were available for 335 patients and 938 samples with at least 10% tumor fraction (Fig. 1a, Additional file 1: Table S1).

### Genomic sequencing data analysis

Sequencing data were processed using the Anduril 2 pipeline [19], following GATK best practices [20] (details in Additional file 1: Supplementary methods). Somatic variants were called using GATK 4.1.9.0 Mutect2, patient-specific germline reference samples and a panel of normals. Variants were annotated with ANNOVAR 20250302 [21], prediction algorithms CGI [22], AlphaMissense [23], PolyPhen2 [24], SIFT [25], and dbscSNV 1.1 [26], and variant databases ClinVar [27] or OncoKB [28]. Copy-number aberrations, tumor-cell fraction, and tumor ploidy were analyzed using GATK version 4.1.4.1 [29] and ASCAT algorithm [30]. GATK-ASCAT output was used for identification of samples with representative tumor-cell fraction (> 10%) and for detection of gene amplifications and large deep deletions. Structural variants were detected using GRIDSS [31] and the HMF toolkit [32]. Additionally, structural aberrations affecting *BRCA1*, *BRCA2*, *RAD51C*, or *RAD51D*, were curated with BasePlayer [33] and SegmentModels Spy [34]. HR-status was defined according to ovaHRDscar [35].

### RNA sequencing data analysis

RNA sequencing data were processed with the SePIA pipeline [36] (details in Additional file 1: Supplementary methods). Amplification-driven gene hyperexpression was detected from a normalized gene expression matrix. Expression of mutant alleles was assessed by force calling the somatic mutations in genome-aligned RNA-sequencing data. Gene fusion events were detected using the nf-core/rnafusion pipeline v. 2.0.0 [37]. Transcriptional footprint analyses of recurrent driver aberrations were performed using the PROGENy [38] and CollecTRI [39] algorithms (details in Additional file 1: Supplementary methods).

### Workflow for identification of credible driver events

The precision oncology workflow employs a systematic, stepwise strategy with stringent criteria to identify credible driver aberrations for oncogenes and tumor suppressor genes (Fig. 1b). To verify the functional impact of genomic aberrations in oncogenes, we confirmed that the relevant alleles were transcriptionally active. Aberrations in tumor suppressor genes were assessed by probability of complete loss of all functional gene copies as a result of a high allele-frequency aberration and a loss-of-heterozygosity.

Briefly, short mutations, copy-number changes, and structural aberrations were called using specific data processing pipelines, as described above, resulting in annotations on variant identity, protein-level consequence, and predicted or known pathogenicity (Additional file 1: Supplementary methods). Short mutations were considered pathogenic if they were previously characterized and included in the ClinVar [27] or OncoKB [28] databases, predicted to be pathogenic based on the consensus of multiple prediction algorithms, or protein-truncating mutations in a tumor suppressor gene. Structural variants disrupting a gene coding region were considered invariably pathogenic, except for such oncogene-associated structural variants where only the first two exons were retained. Multi-site sampling with phylogenetic analysis revealed the behavior of the aberrations along cancer evolution, as described previously [16].

For pathogenic short mutations in tumor suppressor genes, we calculated the expected allele frequency given that the aberration affects all gene copies in cancer cells, using the following formula:

$$\frac{\left(CNmut\times TF\right)}{\left(CNtot\times TF+2\times \left(1-TF\right)\right)}$$

where *CNmut* (number of aberrant gene copies in cancer) and *CNtot* (number of all gene copies in cancer) are given the value of the locus copy number, and *TF* is the tumor fraction, from the GATK-ASCAT copy-number analysis pipeline. For aberrations co-occurring with loss-of-heterozygosity, we assessed the probability of all gene copies being affected by the aberration. With a null hypothesis that all gene copies are affected, we calculated the binomial probability of the observed read count given the sequencing depth. Probability values below 0.01 rejected the null hypothesis and prompted us to assume that the aberration is sub-clonal or heterozygous.

Copy-number losses were interpreted to affect all gene copies, if the estimated copy number of the lowest copy-number segment in the gene was less than 0.5. The number of affected and intact alleles in tandem duplications, translocations, and inversions was calculated as the difference in copy number between the adjacent segments. If the estimated number of intact copies was less than 0.5, all gene copies were assumed to be affected.

For the oncogenes, whose impact arises from aberrant protein function, transcriptomic-level evidence was considered a prerequisite for credibility. Therefore, their credibility was assessed with RNA sequencing data in an aberration type-specific manner, as follows. An amplified gene was required both to be associated with elevated expression overall, as previously reported (CNI > 0.46, CNA transition point > 2 as in [12]), and to have especially high expression in the cancer sample with the amplification (highest 20% in the cohort). A structural change, where the target gene coding region was joined to another genomic region, was considered credible only if an expressed fusion gene was detected and a somatic missense mutation only upon detection of at least five RNA sequencing reads carrying the mutation. Ambiguous cases and splice-aberrations were manually curated by an expert geneticist from BAM files with the help of BasePlayer visualization software [33].

### Assembly of drug target and biomarker data

We first made a comprehensive search for cancer drugs using the Open Targets Platform [40], which resulted in 388 approved and investigational cancer drugs. Then we identified the drug targets followed by delineation of 388 predictive and likely predictive biomarkers (Fig. 1c, Additional file 1: Supplementary methods, Additional file 2: Table S2). Together, these form a comprehensive collection of drug-gene pairs that characterize the actionable landscape of HGSC. To ensure clinical relevance, genomic aberrations were classified using the ESMO scale for clinical actionability of molecular targets (ESCAT) [41], where Tiers IA-C represent clinical actionability in routine care, Tiers II–III denote actionability in clinical trials, and Tiers IV, V, and X represent progressively lower levels of evidence for biomarker–treatment associations.

### Organoid analysis

Patient-derived long-term organoids (PDO) were established from dissociated, fresh tumor tissue samples, verified to match the original tissues, and tested for drug sensitivity as described previously [42]. Briefly, organoids were each maintained in their optimal growth media and screened at two concentrations for each drug, selected based on literature to match physiological doses, alone and in combinations (Additional file 1: Table S3). Medium was replaced every 72–96 h and the organoids were exposed to drugs for 7 days, with drug replenishment after 4 days of treatment, together with the culture medium change. Cell viability was measured after the total of 7 days of drug exposure using automated fluorescent confocal microscopy (Image Xpress Confocal, Molecular Devices) and fluorescent live/dead cell staining with CellTox Green #G8743, Promega, final concentration 1X) and Hoechst 33342 (#B2261, Sigma, final concentration 5 μg/mL) and normalized according to reference conditions of DMSO or 10 μM bortezomib. Cytotoxicities were estimated through image analysis using MetaXpress (Molecular Devices) software (RRID: SCR_016654).

While the drug screen served in identifying promising treatment combinations, the synergy was further tested in the responsive PDO, EOC989_iOme. We titrated decreasing concentrations of carboplatin with either multiselective KRAS(ON) inhibitor RMC-7977 or MEK inhibitor cobimetinib, measured cell viability as above, and quantified the combined effect with Bliss synergy [43].

### Statistical analyses

Associations were tested with two-sided Fisher’s exact test. Aberration homogeneity in cancer cells was assessed at loci of full loss-of-heterozygosity using binomial probability and null hypothesis that all gene copies are affected, as described above.

## Results

**Fig. 2:**
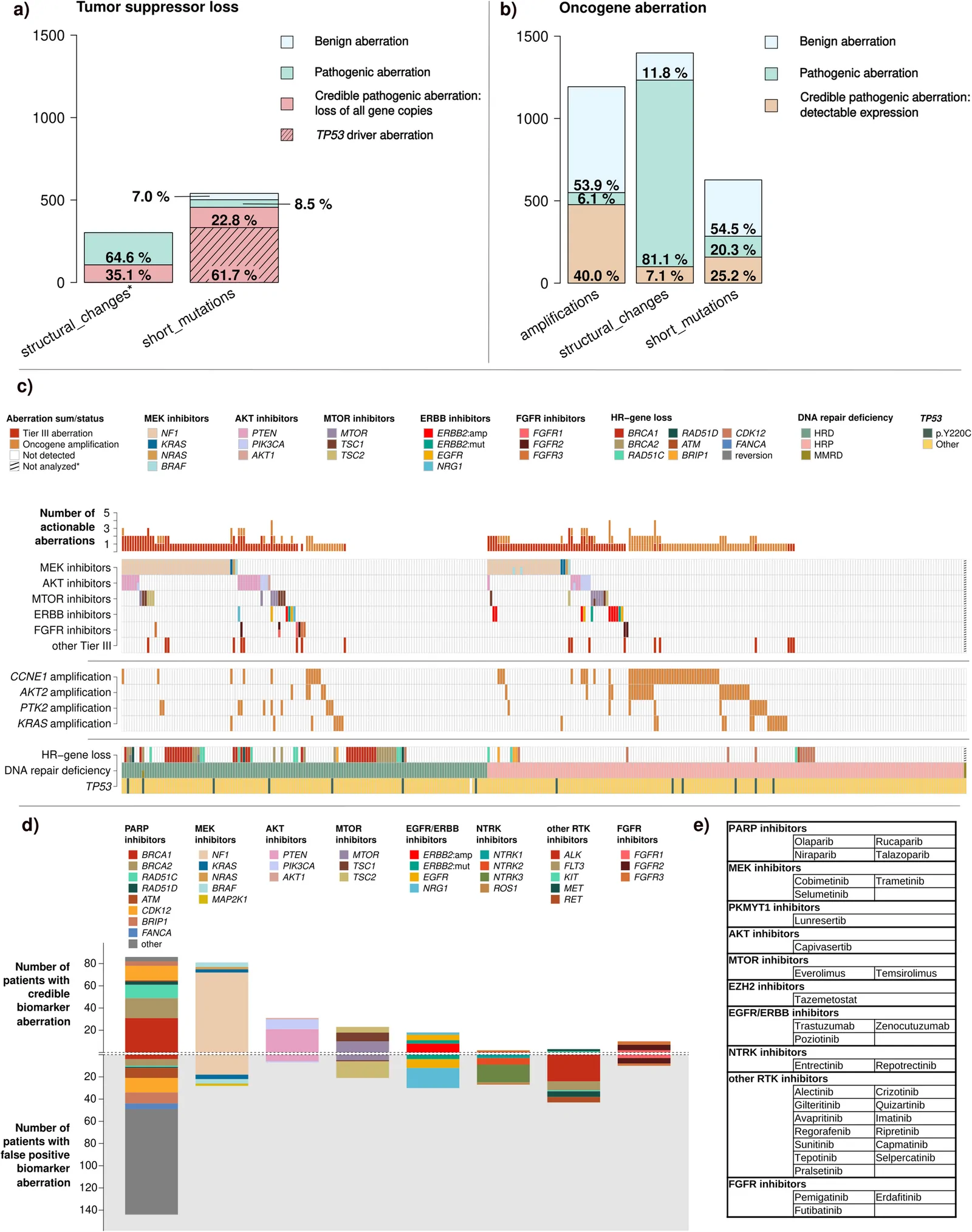
Credible actionable aberrations. Counts of different aberration types, including point mutations, structural variants, and copy-number alterations affecting **a**) tumor suppressor genes or **b**) oncogenes (including all genes encoding drug target proteins). All aberrations affecting the coding sequences of the selected genes, detected in any of the analyzed cancer samples were counted once per a patient. DNA–RNA integration was applied to genes with presumed gain-of-function mechanisms to evaluate functional activity of oncogenic events, whereas quantitative DNA analysis was used for tumor suppressor genes to identify alterations causing complete loss of gene function. * Structural aberrations in tumor suppressor genes include one TP53 driver aberration. **c** Oncoplot illustrating the recurrent, credible, actionable aberrations classified as ESCAT Tier II–IV. Tier II–III aberrations were detected in 40% of patients (top rows: aberrations grouped by associated drug and color-coded by affected gene). Recurrent amplifications of wild-type oncogenes (Tier IVB) are summarized in the middle rows, and HRD-associated genes, mutational signatures, and *TP53* mutations in the bottom rows. * Aberrations affecting biomarker genes were not analyzed for the hypermutated cancer with mismatch-repair deficiency. **d** Evaluation of nominally pathogenic genomic alterations for expression of mutated alleles, hyperexpression of amplified genes, or complete loss of all gene copies reclassified more than half of the aberrations as false positives (grey area). False-positive aberrations were identified across all analyzed genes and drug categories. **e** Representative drugs corresponding to the gene-specific categories are listed in the accompanying table, including at least one approved drug per a target gene

We performed systematic evaluation of somatic aberrations in 388 drug target and biomarker genes with an integrated omics analysis using tumor sequencing data of 938 samples from 355 patients with HGSC enrolled in the DECIDER study (Fig. 1a). The workflow consisted of three major phases as follows: aberration discovery in the DNA sequencing data, assessment of the aberration’s pathogenicity and impact on protein structure, and functional evaluation with distinct criteria for tumor suppressors and oncogenes (Fig. 1b). Aberrations in tumor suppressor genes fulfilled the functional criterion when all gene copies in cancer cells were affected. Aberrations in oncogenes were required to produce an aberrant transcript and amplifications to cause transcript hyperexpression for functional validation. Thus, the analysis enabled stepwise prioritization of all aberrations of interest as shown in Fig. 2.

### Actionable landscape of HGSC

In tumor suppressor genes, we identified 540 short mutations and 302 structural aberrations (Fig. 2a). The majority of short mutations mapped to *TP53*, consistent with the near-universal prevalence of *TP53* mutations in HGSC [13, 44]. Beyond *TP53* mutations, 73% of the pathogenic short mutations and 35% of the structural aberrations resulted in complete loss of gene function.

For oncogenes, structural aberrations represented the most prevalent aberration class (*n* = 1398) followed by amplifications of wild-type genes (*n* = 1193) and short mutations (*n* = 627) as shown in Fig. 2b. Most structural aberrations showed no evidence of functional impact at mRNA level, reducing their relevance as predictive biomarkers. In contrast, about half of the short mutations were expressed. Notably, gene amplifications frequently resulted in marked overexpression of the affected genes, consistent with prior evidence highlighting the central role of copy-number alterations in HGSC [12, 13].

To assess clinical relevance of the genomic aberrations, we matched them to ESCAT tiers as shown in Fig. 2c. Currently, HRD and PARP inhibitors are the only Tier IA combination of a biomarker and therapy for patients with HGSC. In our cohort, we detected HRD in 145 patients (43%) and 42% of them had causal mutations in *BRCA1/2* (*n* = 49) or *RAD51C/D* (*n* = 12). High mutational burden, a Tier IC biomarker for immune checkpoint inhibitor therapy, was detected in one patient, who had mismatch-repair deficiency caused by an *MSH6* mutation.

Tier II–IV drug-gene pairs represent the unexploited potential of precision oncology in HGSC. In our cohort, approximately half of the patients (*n* = 136, 41%) harbored at least one functionally credible Tier II or III aberration with potential for off-label drug use (Fig. 2c, Additional file 3: Table S4, Additional file 4: Table S5). Notably, 10% harbored more than one actionable alteration, including three patients with three aberrations and 34 patients with two. Tier IIIA loss of *NF1* was the most prevalent genomic event (*n* = 72, 21%), suggesting benefit from the MEK inhibitors. Other prominent targetable categories were aberrations associated with AKT (*n* = 29, 9%), MTOR (*n* = 22, 7%), or ERBB2 (*n* = 17, 5%) inhibitors. In general, credible Tier II–III aberrations were detected in both HRD and HR proficient (HRP) tumors with equal frequency. However, both *NF1* aberrations (OR: 2.33; 95% CI: 1.33–4.14; *p* = 0.002) and *PTEN* loss-of-function events (OR: 4.56; 95% CI: 1.55–16.3; *p* = 0.002) were enriched in HRD tumors. Notably, the actionable variants were not recurrent hotspot mutations but unique genomic changes.

### False-positive actionable variants are minimized by integrated DNA-RNA analysis

We then characterized to what extent the presence of Tier I-III aberrations implies clinical actionability. We identified 642 nominally pathogenic aberrations in 57 Tier I-III genes and 274 patients. Strikingly, less than half (*n* = 270, 42%) of these aberrations met the criteria for credible alterations, *i.e.,* functional validation with RNA data (oncogenes) or complete loss of all functional gene copies (tumor suppressor genes), as shown in Fig. 2d. The proportion of false-positive findings varied between biomarker genes. For instance, 80% of deleterious *NF1* aberrations caused full loss of the gene function, suggesting clinical relevance. Similarly, most aberration in *PTEN* (78%), and *MTOR* (67%) were found credible.

In contrast to *NF1*, *PTEN* and *MTOR*, pathogenic missense mutations or translocations in receptor tyrosine kinase genes, such as *ALK*, *FLT3*, *KIT*, *MET*, *RET*, *ROS1*, *NTRK1-3*, were predominantly non-expressed passenger events (Fig. 2d). This limits their clinical relevance, which is also supported by their borderline expression in the tissue of origin, the fallopian tube (GTEx v10, Additional file 1: Fig. S1). Notably, false-positive biomarkers were also discovered for PARP inhibitors, including primarily subclonal or heterozygous mutations in secondary HR-pathway genes (*ATM*, *ATR*, *BARD1*, *BRIP1*, *CDK12*, *CHEK1*, *CHEK2*, *FANCA*, *FANCL*, *MLH1*, *MRE11*, *NBN*, *RAD51B*, and *RAD54L*) and occasional heterozygous alterations in *BRCA1/2*. Accordingly, these aberrations showed no significant association with HRD (OR:1.13; 95% CI: 0.71–1.80; *p* = 0.58).

Importantly, even hotspot mutations were occasionally rendered as false-positive biomarkers after functional evaluation. For example, *BRAF*:p.D594N was detected in one of three tumor samples from EOC438. Phylogenetic analysis indicated that the mutation was confined to a sample-specific subclone in the left-ovarian sample, which had high tumor cellularity (tumor fraction 0.95, Additional file 1: Fig. S2 a). However, no mutant allele reads were detected in RNA sequencing, whereas the wild-type *BRAF* expression was supported by 40 reads. In the same RNA sample, several other subclone-specific somatic aberrations were expressed, supporting adequate RNA-based detection. Similarly, *EGFR*:p.G796R was detected in one high-quality tumor sample from EOC206 (tumor fraction 0.87, Additional file 1: Fig. S2 b), but RNA analysis showed complete repression of *EGFR* expression, with neither wild-type nor mutant allele reads detected in mature mRNA. Sample identity and RNA informativeness were supported by expression of HGSC-characteristic genes, including *ERBB2*, and multiple private somatic aberrations. These examples illustrate that using genomic biomarkers without functional evaluation may lead to inclusion of non-expressed, and therefore likely non-actionable, hotspot mutations.

### Amplifications of wild-type oncogenes are credible driver events

Next, we explored the impact of Tier IVB wild-type proto-oncogene amplifications, which are characteristic to HGSC. The most recurrent amplifications supported by transcriptional overexpression were *CCNE1* (*n* = 56, 17%), *AKT2* (*n* = 31, 9%), *PTK2* (*n* = 22, 7%), and *KRAS* (*n* = 22, 7%) as shown in Fig. 2c. These amplifications were enriched in HRP tumors (OR: 2.78; CI: 1.64–4.80; *p* = 0.00005), with *AKT2* amplifications occurring almost exclusively in HRP cases and *PTK2* amplifications showing comparable frequencies in HRP and HRD tumors.

**Fig. 3:**
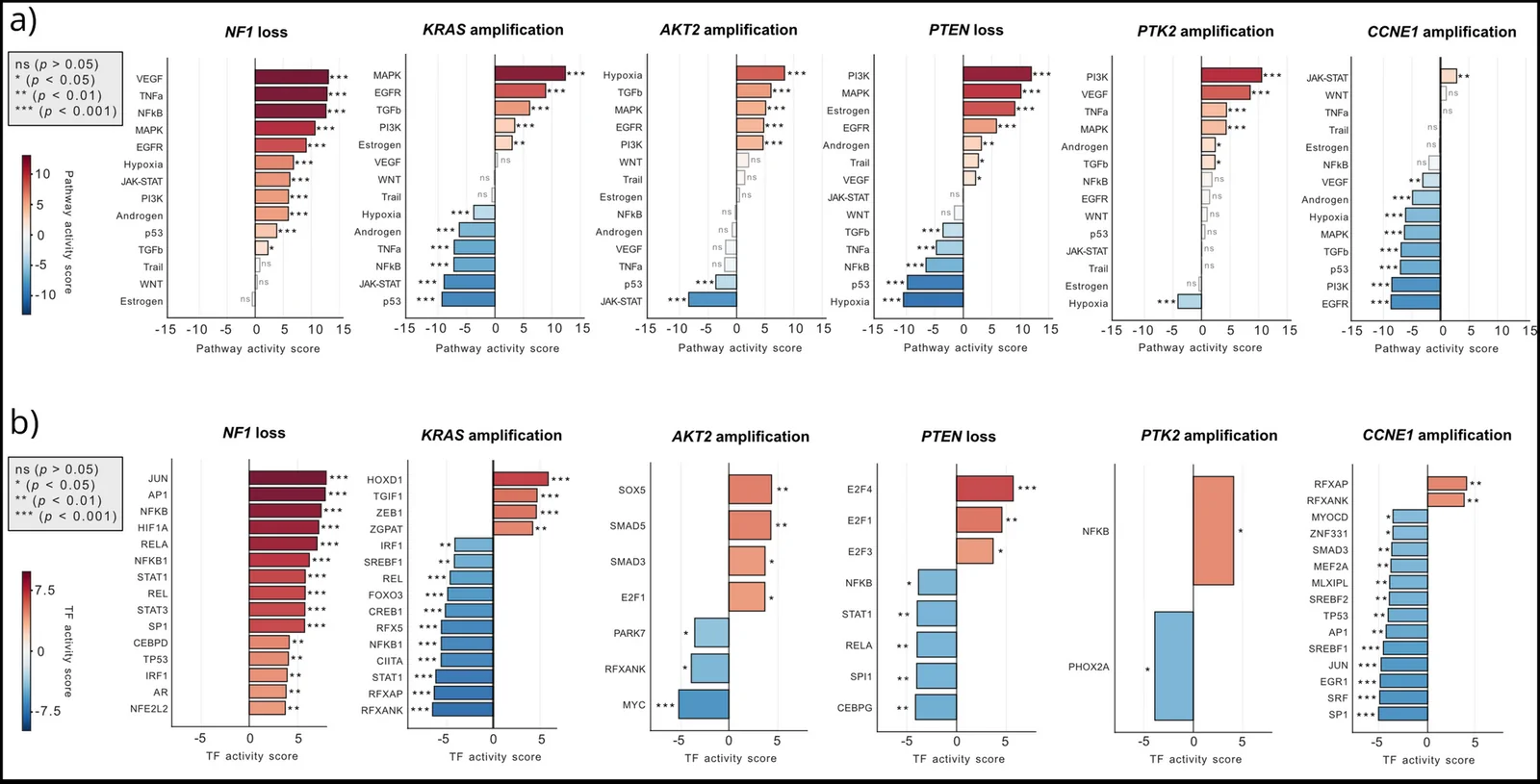
Transcriptional footprints of recurring driver aberrations. **a** Differential pathway activity inferred with PROGENy from aberration-specific differential expression contrasts comparing samples with the indicated aberration against samples without that aberration, while adjusting for the status of the remaining recurrent aberrations. Positive scores indicate higher inferred pathway activity in aberrant samples [38]. **b** Differential transcription factor activity inferred with CollecTRI from the same contrasts using signed TF-target regulons [39]. Only significant associations (after correction for multiple testing) are shown

Expression analysis showed that *CCNE1*, *AKT2*, *PTK2*, and *KRAS* amplifications cause hyper-expression of the wild-type alleles. Moreover, the amplifications represented credible driver events modulating the activity of cancer-associated pathways, each characterized by a distinct transcriptional signature (Fig. 3, Additional file 1: Fig. S3). Amplifications of *AKT2*, *PTK2*, or *KRAS* were linked to aberrant activation of MAPK and PI3K signaling cascades, mirroring loss-of-function events in *NF1* and *PTEN*. Furthermore, amplifications of *AKT2* or *KRAS* triggered elevated activity of TGF-β-dependent signaling and epithelial-to-mesenchymal transition via SMAD-family or homeobox transcription factors, whereas *PTK2* amplification phenocopied NF1 deficiency, showing high activity of TNF-α and NF-κB signaling and their associated transcriptional regulators. *CCNE1* amplification was not associated with perturbed signaling cascades, aligning with its role as a regulator of cell cycle progression.

### Pre-treatment samples are informative for clinical decisions at relapse

To guide optimal sampling strategies for identifying predictive biomarkers at relapse, we analyzed Tier II–III aberrations across multiple anatomical locations before and during therapy. Credible aberrations were detected roughly four times more frequently across all anatomical sites compared with false-positive events (OR: 3.83; CI: 2.61–5.66; *p* = 1.3·10^13^). Among credible aberrations, 74% were present in all sampled anatomical locations, with no significant differences between sites (*p* = 0.47). This implies that credible aberrations with clinical utility are predominantly clonal events.

**Fig. 4:**
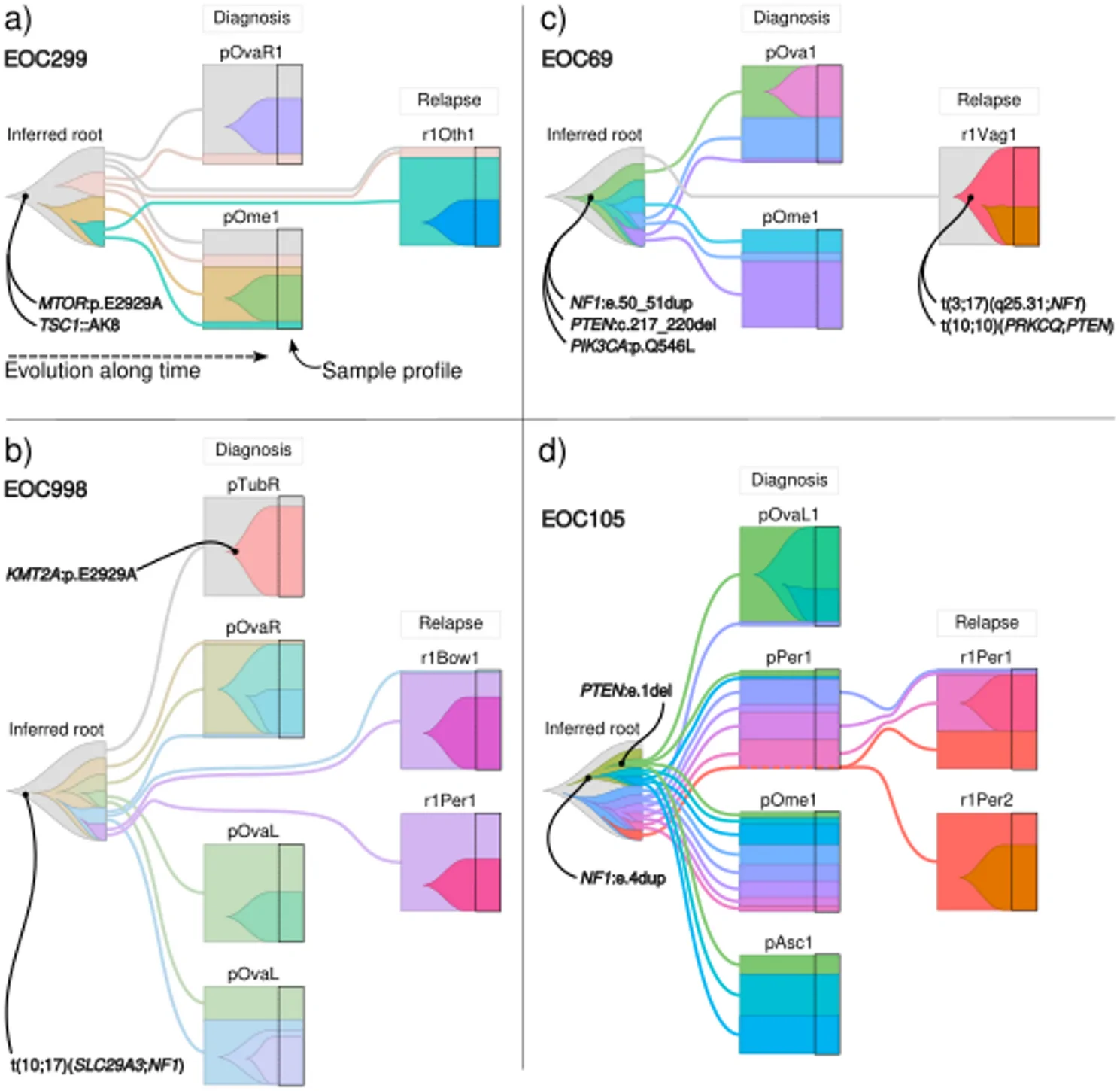
Example cases of longitudinal patterns. Jellyfish plots describing subclonal cell populations across all analyzed samples from the patient. Mutations assigned to the grey area, the root, are present in all subclones, whereas mutations arising in colored subclones are transmitted to all their offspring. A pointed edge on the left of a colored region illustrates the separation of a novel subclone from a parental clone, and the height of the colored region on the right, the proportion of the subclone in the analyzed cancer sample. The actionable aberrations are mapped to their appropriate subclones. **a** Patient EOC299 had clonal aberrations in two treatment-guiding biomarker genes. Consequently, the same aberrations were detected in all primary and relapse samples. **b** Patient EOC998 had a clonal translocation causing full loss of *NF1* detected in all cancer samples, and a site-specific aberration in *KMT2A*, detected merely in the dextral fallopian tube. **c** Patient EOC69 exemplified convergent evolution with non-oncogene addiction to loss of *NF1* and *PTEN*. The PIK3CA missense mutation was seen only in the ovarian sample. **d** Patient EOC105 had sub-clonal alterations in *NF1* and *PTEN*. At diagnosis, the allele frequencies varied by the sample sub-clonal composition, so that the sub-clonality was detectable only in the omental and peritoneal samples. Neither of the aberrations was detected in relapse samples, representing an evolutionary branch that was not captured in the diagnostic samples. Interactive Jellyfish visualization is available in https://hautaniemilab.github.io/jellyfish/, and is described in Lavikka et al. 2025 [45]. (abbreviations used in tumor samples: p = primary debulking surgery, r = palliative relapse operation, Ova = ovary, Ome = omentum, Oth = other cancer site, Per = peritoneum, Asc = ascites, Tub = fallopian tube, Bow = bowel, Vag = vagina, R = dextral, L = sinistral)

To further explore the longitudinal stability of credible aberrations, we used paired primary and relapse samples from 21 patients with 34 Tier II–III aberrations. Most of the aberrations (71%) were identical in primary and relapse samples, indicating early emergence and subsequent clonal stabilization, like in an example patient illustrated in Fig. 4a.

Further exploration of the discordant aberrations between primary and relapse samples revealed that subclonality is often detectable at diagnosis as shown in Fig. 4b-d. Briefly, two patients had tubo-ovarian-specific mutations that were not present in relapses, whereas the treatment-naïve metastasis samples carried the same the actionable biomarkers as the relapse sample (Fig. 4b-c). Interestingly, one of these and another patient harbored converging subclonal aberrations with the same gene-level consequence, suggesting strong selective pressure to maintain activation of key growth signaling pathways (Fig. 4c). Furthermore, four patients had actionable aberrations present in both tubo-ovarian and metastatic samples, but low variant allele frequencies in the metastatic samples revealed the subclonality (Fig. 4d).

### Patient-derived organoids suggest MEK- and KRAS-targeting therapies effective in HGSC

**Fig. 5:**
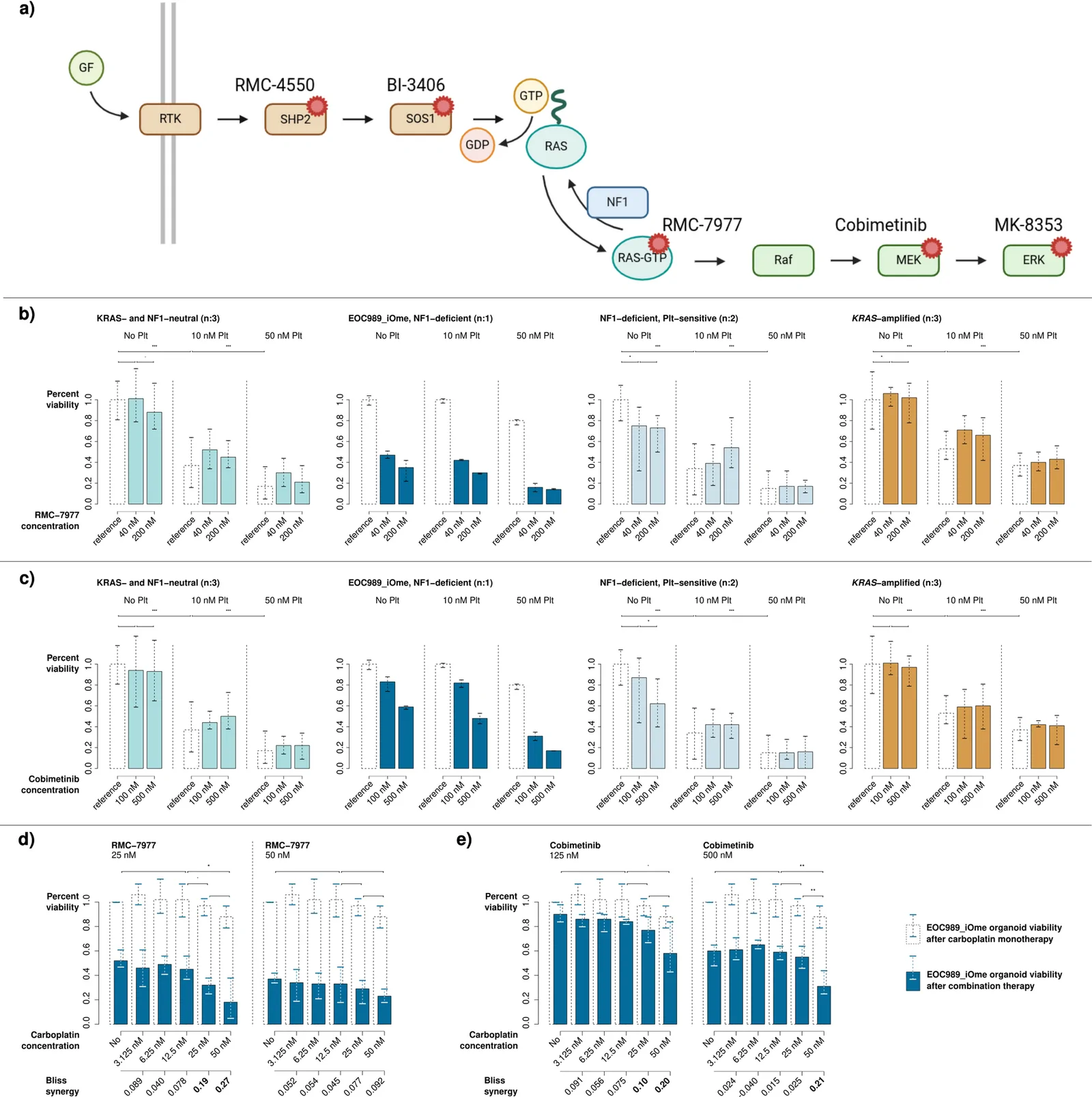
Drug sensitivity of patient-derived organoids. **a** Schematic presentation of the RAS-MAPK signaling cascade. The pathway components targeted in the drug screen in organoids are marked with red asterisks. (GF: growth factor, RTK: receptor tyrosine kinase) Drug response measured as normalized percent viability after treatment with **b**) KRAS inhibitor (RMC-7977) or **c**) MEK-inhibitor (cobimetinib) at two concentrations as monotherapy and in combination with two carboplatin concentrations. The plots include mean and range over organoids from different patients, each measured in two replicates. Significance bars are shown for comparisons of increasing monotherapy doses for experimental groups consisting of multiple organoids. Synergy between carboplatin and targeted therapy with **d**) KRAS inhibitor or **e**) MEK inhibitor was observed when combining sub-lethal concentrations of the drugs. The plots include mean and range over two technical and two biological replicates. Significance bars are shown for comparisons of combination therapies (dark-blue bars)

To investigate the therapeutic potential of NF1 deficiency, which was the most recurrent Tier III biomarker, we performed a focused drug screen using patient-derived organoids. Organoid cultures were established from three patient cases harboring deleterious and homogeneous *NF1* aberrations, three cases with *KRAS* wild-type amplification, and three reference cases lacking driver events in the RAS-MAPK pathway. To inhibit both up- and downstream components of the pathway, we used five inhibitors as monotherapies and in combination with other compounds (Fig. 5a, Methods).

Monotherapy with the investigational, multiselective KRAS(ON) inhibitor RMC-7977 demonstrated the strongest activity against NF1-deficient PDOs, exceeding the effect of the MEK inhibitor cobimetinib, which is a Tier III drug for NF1-deficient tumors (Fig. 5b,c; Additional file 5: Table S6). Sensitivity to both RMC-7977 and cobimetinib was specific to NF1 deficiency, as no reduction in viability was observed in the reference PDOs. In contrast, compounds targeting upstream (SHP2 or SOS1) or downstream (ERK) components of the pathway had little impact on PDO growth regardless of mutation status (Additional file 5: Table S6).

RMC-7977 or cobimetinib re-sensitized the platinum-resistant PDO to carboplatin (Fig. 5d,e, Additional file 5: Table S7). In contrast, the two platinum-sensitive NF1-deficient PDOs showed no additional benefit from the combination. Instead, the response to the combination mimicked the response to carboplatin alone (Fig. 5b,c), a pattern known as single-agent dominance, which is frequently seen in vitro for clinically effective treatment combinations [46]. Thus, these findings suggest that platinum re-challenge combined with targeted KRAS or MEK inhibition warrants further studies as a therapeutic strategy for patients with NF1-deficient HGSC.

Unlike the NF1-deficient models, organoids with *KRAS* amplification were resistant to RMC-7977, cobimetinib, and all other compounds tested (Fig. 5b,c; Additional file 5: Table S6), aligning with recent reports suggesting that drugs acting on the inactive, GDP-bound KRAS would be superior to KRAS(ON) inhibitors for cancers with wild-type *KRAS* amplification [47].

## Discussion

Recognizing that false-positive calls remain a major obstacle to effective precision oncology trials [2, 48], we developed an integrated whole-genome and transcriptome workflow designed to reliably identify clinically actionable aberrations. In the prospective, observational DECIDER study, we detected credible ESCAT Tier II–III alterations with potential therapeutic relevance in approximately 40% of 335 patients. Notably, 58% of all variants initially classified as pathogenic were found to be false positives. For example, mutations in secondary HR-pathway genes rarely resulted in complete loss of gene function or a corresponding HRD phenotype, and Tier IC *NTRK1–3* alterations were credible in only a minority of cases. These findings indicate that many previously proposed predictive biomarkers lack true functional relevance. Thus, trials that enroll patients based on presumed pathogenic alterations in target genes are likely to be substantially affected by false-positive biomarkers, diminishing the observable benefit of the therapies under investigation.

Multi-modal evaluation of treatment-guiding aberrations shifts the focus from detection alone to a more nuanced assessment of activity and subclonality. To mimic clinical decision-making based on all available evidence, we applied predefined criteria for biomarker credibility, although alternative thresholds may also be clinically justifiable. Notably, several subclonal aberrations were evaluated as functionally credible: RNA analysis sensitively detected expressed oncogenic mutations in subclones and low-cellularity samples, and homogeneous tumor suppressor loss was occasionally restricted to a single sample. Although targeting clonal aberrations is expected to yield stronger responses, targeting subclonal driver aberrations may still provide clinical benefit in metastatic HGSC [49].

Longitudinal analysis of paired primary and relapse tumors revealed that most actionable aberrations were clonally stable and present across multiple sites. This suggests that true driver aberrations underlying oncogene addiction are established early in tumor evolution, consistent with the current evolutionary models [6, 50]. Therefore, archival diagnostic samples could reliably capture most clinically relevant driver events in HGSC. However, the biomarker detection should be based on metastatic samples, instead of the tubo-ovarian sites that are enriched with private mutations [16].

We characterized the actionable landscape in HGSC and identified several recurring credible targetable aberrations. For example, *NF1* or *PTEN* loss were often found credible and supported by strong impact on cell transcriptional programs. Furthermore, wild-type amplifications of oncogenes including *AKT2*, *PTK2*, and *KRAS* were identified as credible driver events in a substantial proportion of patients with HGSC, highlighting the importance of preclinical studies to assess their potential therapeutic vulnerabilities.

We explored the translational potential of NF1 deficiency, which was the most recurrent actionable aberration, with a focused drug screen targeting RAS–MAPK pathway components in PDOs stratified by mutation status. PDOs harboring *NF1* loss-of-function aberrations displayed marked sensitivity to KRAS- and MEK-inhibition, earlier proven effective as monotherapies against NF1-deficient neurofibromas [51, 52]. Although MEK inhibitors have shown limited efficacy as monotherapy in HGSC [53, 54], unlike in low-grade serous carcinoma [55], our data suggest that RAS-MAPK pathway inhibition may still provide therapeutic value in combination regimens. Indeed, in a platinum-resistant NF1-deficient PDO, inhibition of MEK or KRAS effectively re-sensitized the cells to carboplatin, aligning with the findings from a recent study on an NF1-deficient ovarian cancer cell line [56].

The observational design of the real-world DECIDER study presents both strengths and limitations. It supports unbiased and comprehensive identification of actionable aberrations but precludes direct evaluation of biomarker-specific treatment responses. Accordingly, dedicated clinical trials are needed to evaluate the therapeutic benefit of the proposed combinations. We note that several of the findings presented here were reviewed by the molecular tumor board at Turku University Hospital and informed the referral of three patients to the biomarker-guided FINPROVE clinical trial [57].

## Conclusions

Our multi-omics workflow provides a generalizable strategy to overcome a major barrier to precision oncology by minimizing the number of false-positive predictive biomarkers that otherwise could reduce the treatment efficacy of targeted therapies in clinical trials. While whole-genome sequencing is only entering the clinical practice, our evaluation principles are applicable also to targeted gene-panels. We comprehensively characterized the actionable landscape in HGSC and identified clinically relevant vulnerabilities that could expand precision-therapy options in over 40% of patients. Furthermore, using PDOs, we provide preclinical evidence supporting combination therapy of carboplatin and targeted drugs for HGSC patients with chemoresistant disease.

## Supplementary Information

Additional file 1. Supplementary methods; Fig. S1, Baseline expression of specific drug target genes in fallopian tube tissue as in the GTEx dat; Fig. S2, Profiles of tumor subclones for patients EOC438 a) and EOC206 b), harboring inert hotspot mutations of *BRAF* and *EGFR*, respectively; Fig. S3, Results from the CORNETO analysis of differential gene expression; Table S1, Clinical characteristics of the 335 study patients; Table S3, Drug concentrations; STROBE checklist.

Additional file 2. Table S2, Drugs and biomarkers.

Additional file 3. Table S4, Somatic aberrations per patient.

Additional file 4. Table S5, Sample-level breakout of somatic aberrations.

Additional file 5. Table S6, Drug screen results; Table S7, Drug synergy analysis.

## Data Availability

Source files for sequencing data are made public in the European Genome-Phenome Archive. Whole-genome sequencing data are available from EGAS00001006775 (https://ega-archive.org/studies/EGAS00001006775) and RNA sequencing data from EGAS00001004714 (https://ega-archive.org/studies/EGAS00001004714). Three of the nine tested organoids are deposited in the Auria Biobank (Turku, Finland). Data used for panel of normals in mutation calling was partly obtained from The Cancer Genome Atlas managed by the NCI and NHGRI with dbGap accession number phs000178 (https://ncbi.nlm.nih.gov/projects/gap/cgi-bin/study.cgi?study_id=phs000178.v9.p8). GTEx v10 data (gene_tpm_v10_fallopian_tube.gct.gz) were obtained from: the GTEx Portal (https://www.gtexportal.org/home/downloads/adult-gtex/bulk_tissue_expression) on 2025-06-02. All analyses and data processing were performed with open-source, published software, as described above. Custom analysis scripts are provided in https://github.com/HautaniemiLab/Actionable. The workflow is currently being integrated into an open-source platform of clinical decision making https://github.com/oncodash/oncodash. [58–60].

## Abbreviations

ESCAT: ESMO scale for clinical actionability of molecular targets
HGSC: Ovarian high-grade serous carcinoma
HRD: Homologous recombination deficiency/deficient
HRP: Homologous recombination proficiency/proficient
NACT: Neoadjuvant chemotherapy
PDO: Patient-derived organoid
PDS: Primary debulking surgery
