# Supplementary material for: Multi-modal data integration reveals functionally credible predictive biomarkers in ovarian cancer
Source: Genome Med. 2026 Jul 3;18:133. doi: 10.1186/s13073-026-01706-x (PMC13579925; doi:10.1186/s13073-026-01706-x)
Supplement: Supplementary file 1 [file 13073_2026_1706_MOESM1_ESM.pdf]

Multi-Modal Data Integration Reveals Functionally Credible Predictive Biomarkers in Ovarian Cancer

Taru A. Muranen[1], Andreas Hainari[1], Daria Afenteva[1], Wojciech Senkowski[2], Jaana Oikkonen[1], Johann Dreo[3], Kyriaki Driva[2], Susanna Holmström[1], Veli-Matti Isoviita[1,4], Alexandra Lahtinen[1], Kari Lavikka[1], Yilin Li[1], Marta Lovino[5], Ilari Maarala[1], Giovanni Marchi[1], Francesca Miccolis[5], Giulia Micoli[1], Matthieu Najm[3], Ann-Christin Ostwaldt[1], Pablo Rodriguez-Mier[6], Jenni Söderlund[4], Sakari Hietanen[4], Anni Virtanen[7], Julio Saez-Rodriguez[6,8], Elisa Ficarra[5], Benno Schwikowski[3], Erika Alanne[9], Krister Wennerberg[2], Sampsa Hautaniemi[1,*], Johanna Hynninen[4,*]

1. Research Program in Systems Oncology, Research Programs Unit, Faculty of Medicine, University of Helsinki, Helsinki, Finland
2. Biotech Research & Innovation Centre, University of Copenhagen, University of Copenhagen, Copenhagen, Denmark
3. Computational Systems Biomedicine Lab, Institut Pasteur, Universite Paris Cité, Paris, France
4. Department of Obstetrics and Gynecology, University of Turku and Turku University Hospital, Turku, Finland
5. Department of Engineering, Enzo Ferrari, University of Modena and Reggio Emilia, Modena, Italy
6. Institute for Computational Biomedicine, Heidelberg University, Faculty of Medicine, and Heidelberg University Hospital, Heidelberg, Germany
7. Department of Pathology, University of Helsinki and HUS Diagnostic Center, Helsinki, Finland
8. European Bioinformatics Institute (EMBL-EBI), Hinxton, Cambridgeshire, UK
9. Department of Oncology, Turku University Hospital and Western Finland Cancer Center, Turku, Finland

* Corresponding authors:

Sampsa Hautaniemi, sampsa.hautaniemi@helsinki.fi

Johanna Hynninen, mijohy@utu.fi

Supplementary methods

Sample processing

Fresh frozen tumor samples were retrieved from PDS, IDS, pre-NACT laparoscopy, drained ascites or pleural fluid, or occasional palliative relapse operations, as previously described [1]. The tumor samples were snap-frozen in liquid nitrogen without delay in the surgical unit.

DNA and RNA were extracted from the fresh-frozen tumor samples and submitted to genome-wide sequencing with either DNBSEQ (BGISEQ-500 RRID: SCR_017979 or MGISEQ-2000 RRID: SCR_017980), HiSeq X Ten (RRID: SCR_016385), or NovaSeq 6000 (RRID: SCR_016387), as previously described [2,3]. Patient-specific germline references were retrieved by whole-genome sequencing of DNA extracted from peripheral blood samples.

Genomic sequencing data analysis

Sequencing data preprocessing

Sequenced read data were processed using Anduril 2 as previously described [1,4] with additional adapter read filtering. Briefly, we filtered read pairs where the read or mate led with CGGTGGT/CGTATGC exactly or CGGTGGTCG/CGTATGCCG with one mismatch from samples where at least 0.1% of the reads would be filtered. Read data were then trimmed using Trimmomatic 0.32 [5] and aligned against genome build GRCh38.d1.vd1 with BWA-MEM 0.7.12-r1039 [6]. This was followed by duplicate marking using Picard 2.6 [7] and base quality score recalibration using GATK 3.7 [8]. We proceeded with normal samples with less than 10% and tumor samples with less than 3% contamination as estimated using GATK 4.1.9.0 and additionally VerifyBamID 2 [9] for normals.

Variant calling and annotation pipeline

Somatic variants were called in joint calling mode using GATK 4.1.9.0 Mutect2 against a patient-specific germline reference sample and a panel of normals as previously described [1]. We applied the additional filters used in signature analysis and further excluded multi-nucleotide variants if any of their nested SNVs were uncallable by Mutect2. Furthermore, for batches highly enriched with C>T artefacts, at GCA/GCC and exhibiting orientation bias, we applied additional patient-specific filtering using ROQ annotations from customized runs of GATK FilterMutectCalls, involving sample-wise recomputed F1R2 counts and subsequent orientation models of tumor samples from affected batches. For driver event analysis, we also dropped variants flagged by GATK FilterAlignmentArtifacts. Furthermore, for batches highly enriched with C>T artefacts, at GCA/GCC and exhibiting orientation bias, we applied additional patient-specific filtering using ROQ annotations from customized runs of GATK FilterMutectCalls, involving sample-wise recomputed F1R2 counts and subsequent orientation models of tumor samples from affected batches. Germline variants were called via joint genotyping using GATK 4.1.9.0. Allele dosage of germline variants in tumor samples was

estimated patient-wise by forced calling using Mutect2 in joint calling mode. We applied variant annotations using ANNOVAR 20250302 [10], and used MANE v1.4 [11] for gene-based annotations.

The pathogenicity of missense aberrations was estimated with CGI [12], AlphaMissense [13], PolyPhen2 [14], and SIFT [15]. If an aberration was previously characterized and present in the curated ClinVar [16] or OncoKB [17] databases, this annotation overruled in silico predictions. In other cases, pathogenicity was based on consensus calling of the predictor algorithms, so that a missense was considered potentially pathogenic if it was called oncogenic by CGI output or deleterious by two of the remaining three algorithms. The effect on splicing was estimated with dbscSNV 1.1 [18] and aberrations with ADA or RF score exceeding the threshold value 0.95 were considered potential splice-aberrations. Insertions, deletions, and nonsense mutations were always considered potentially pathogenic. Frameshift and nonsense aberrations were evaluated further only for tumor suppressor genes.

We determined HRD status based on ovaHRDscar, which is used in HGSC clinical diagnostics in Finland [19].

Copy-number calling

We performed copy-number segmentation using GATK version 4.1.4.1 [20], and estimated tumor purity, ploidy, allele-specific copy numbers and purified logR values with a reimplementation of the ASCAT algorithm [21], as detailed in Lavikka, 2024 [22].

GATK-ASCAT output was used for detection of an amplification, using a threshold value of purified log-ratio high than 2.5, corresponding to a gene copy number approximately three times the cancer cell ploidy, and estimated copy number of at least eight. A genomic segment was considered deleted if the purified logR was lower than –2. Deletions were considered unreliable in cancer samples with tumor fraction below 0.2 and these were not used in further analyses.

Structural aberration calling

Structural variant calling was carried out using GRIDSS [23] in combination with the HMF toolkit [24]. Somatic variants were filtered with GRIPSS, using a panel of normals from DECIDER and Dutch population blood samples, and excluding regions listed in the ENCODE and DECIDER blacklists, as previously explained [2]. GRIDSS output was considered only for the detection of structural aberrations, not for the detection of amplifications or large deletions. The gene-level consequences of the somatic structural aberrations were annotated based on transcripts in GENCODE v47, using custom code, and only aberrations causing a change in a gene coding region were retained for further analyses.

Variant curation

The characterization and targeted exploration of DNA alterations involved joint visualization of mutations, copy-number segments, and structural aberrations at cohort and patient levels using GenomeSpy [22]. Subsequent data management, analyses, and visualizations were performed in R environment for statistical computing version 4.2.1 [25]. Cancer subclonal profiles, as published earlier [1], were visualized with Jellyfish plots, produced with jellyfisher R package [26].

RNA sequencing data analysis

RNA processing, decomposition, and normalization

RNA sequencing data were processed with Anduril 2 using the SePIA pipeline [27], including Trimmomatic [5], STAR aligner [28], and eXpress [29]. Tumor cell-specific expression was decomposed with PRISM [30]. For estimation of an amplification-driven gene hyperexpression, the expression matrix was normalized with variance stabilizing transformation, using the R library DESeq2 [31].

Single-nucleotide variant calling

Patient-specific somatic missense variants were force-called in all RNA sequencing samples from the same patient with the GATK function ASEReadCounter. Variant alleles with at least five supporting sequencing reads in RNA data were considered expressed.

Fusion analysis

Gene fusion events were detected in RNA-seq data using the nf-core/rnafusion pipeline v. 2.0.0 [32], which integrates Arriba v2.2.1 [33], STAR-Fusion v1.10.1 [34], Pizzly v0.37.3 [35], and Squid v1.5 [36] with default parameters.

To ensure the identification of biologically relevant and transcribed fusion transcripts with the potential to exert specific effects, stringent criteria were applied. Specifically, only gene fusions supported by a minimum of five reads were included in the analysis. Fusion transcripts consisting of opposite orientation fusion partners of gene 3’ ends were excluded, as well as fusions where both partners were out-of-frame. Fusion transcripts involving only one protein-coding gene in open reading frame were annotated as truncated transcripts coding an abnormal protein. Intra-genic fusions were annotated for the reading frame status and used in validation of intra-genic large deletions and duplications.

Transcriptional footprint analysis

The transcriptional impact of the recurring driver aberrations in *NF1*, *CCNE1*, *PTEN*, *AKT2*, *PTK2*, and *KRAS* was assessed using footprint-based pathway and trascription factor activity inference. Pathway activity was analyzed using PROGENy, which includes curated transcriptional outputs for 14 pathways [37]. Transcription factor activity was inferred with CollecTRI [38] across 43,175 transcription factor-target interactions using the Python package

decoupleR [39]. For both PROGENy and CollecTRI analyses, multiple-testing corrections were performed separately for each aberration-specific contrast across all pathways or transcription factor-target interactions.

For the analysis, we selected one representative tumor sample per patient, prioritizing solid tumor samples from metastatic sites with the highest fraction of tumor cells. Analysis of differential expression was conducted with pydeseq2 [40]. Aberration-specific contrasts were obtained from the following multivariable model:

Expression ~ *NF1* + *CCNE1* + *PTEN* + *AKT2* + *PTK2* + *KRAS*

Each covariate represented dichotomous aberration status of the corresponding gene in the sample, as follows: *NF1* normal/loss, *CCNE1* normal/amplified, *PTEN* normal/loss, *AKT2* normal/amplified, *PTK2* normal/amplified, *KRAS* normal/amplified. Thus, for each gene, the contrast compared samples with the indicated aberration against without that aberration, while adjusting for the status of the other recurrent aberrations.

Assembly of drug target and biomarker data

To establish a comprehensive listing of available targeted therapies, we collected 388 approved and investigational cancer drugs from Open Targets [41] and OncoKB [17] databases with their direct physical protein targets (n =352) or genes associated with specific sensitivity (n =36). From the Open Targets Platform release 24.03, we downloaded the following data objects on 2024-05-29: “Drug - indications” and “Drug - mechanism of action”. These datasets were integrated using ChEMBL IDs as a common identifier. To refine our selection, we filtered drugs based on MONDO_0004992, an ontology term specifically describing cancer, and further filtered to exclude anti-microbiological drugs, generic chemotherapies, including all drugs approved prior to the year 1990, withdrawn drugs, and drugs whose development were canceled after Phase I clinical trials. Furthermore, antibody-drug conjugates were excluded, to focus on small molecule targeted inhibitors. OncoKB Version 4.1 data on drugs and biomarker genes and aberrations were downloaded on 2024-12-02.

To enable agnostic mapping of somatic events in cancer, we assembled the drug-target biomarker connections into a logical network, representing the mechanistic relations between the components (Figure 1b, Additional file 2: Table S2). As in the Open Targets Platform, we included in the network all physical protein targets, affected at a comparable concentration scale, and interpreted these as potentially actionable targets. Furthermore, we distinguished between the concept of the physical drug target and the concept of the aberrated biomarker gene. In alignment with the OncoKB database, actionable biomarkers were recorded primarily on a general level, including gene identity and the consequence, either ‘gain’ or ‘loss’, that an aberration in the gene must have to be classified as oncogenic. This approach enabled us to separate the mapping of potential drugs and the prediction of aberration consequence, to carry out a comprehensive analysis of a cancer type with few well-characterized driver aberrations.

Fig. S1. Baseline expression of specific drag target genes in fallopian tube tissue as in the GTEx data.

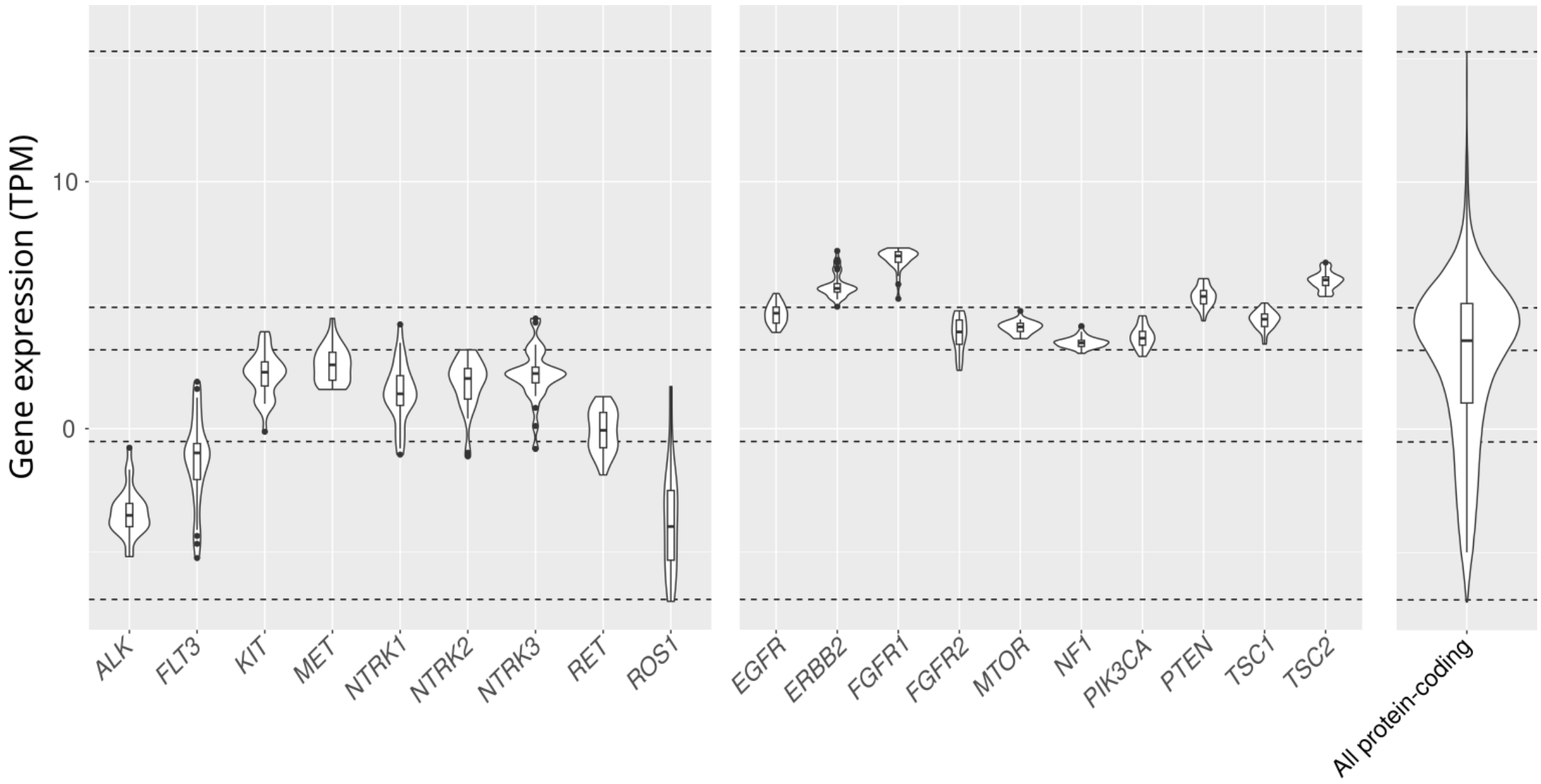

Fig. S2. Profiles of tumor subclones for patients EOC438 a) and EOC206 b), harboring inert hotspot mutations of *BRAF* and *EGFR*, respectively. The *BRAF* and *EGFR* mutations in these cases were sub-clonal and sample-specific passenger aberrations, restricted to the primary site. On the contrary, the *MTOR* mutation detected in metastatic sample of EOC438 was transcriptionally active, suggesting presence of an altered oncogenic protein.

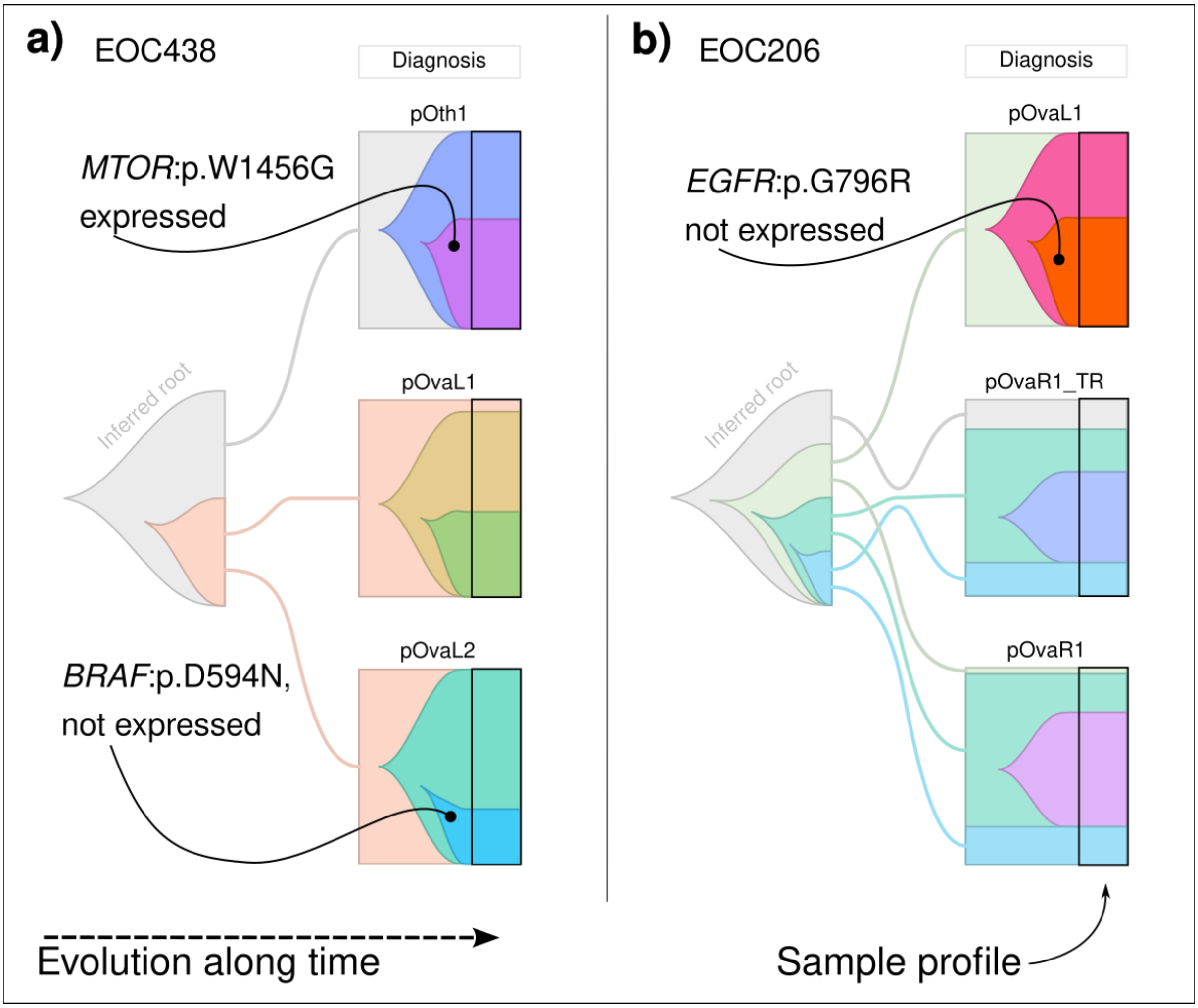

Fig. S3. Results from the CORNETO analysis of differential gene expression.

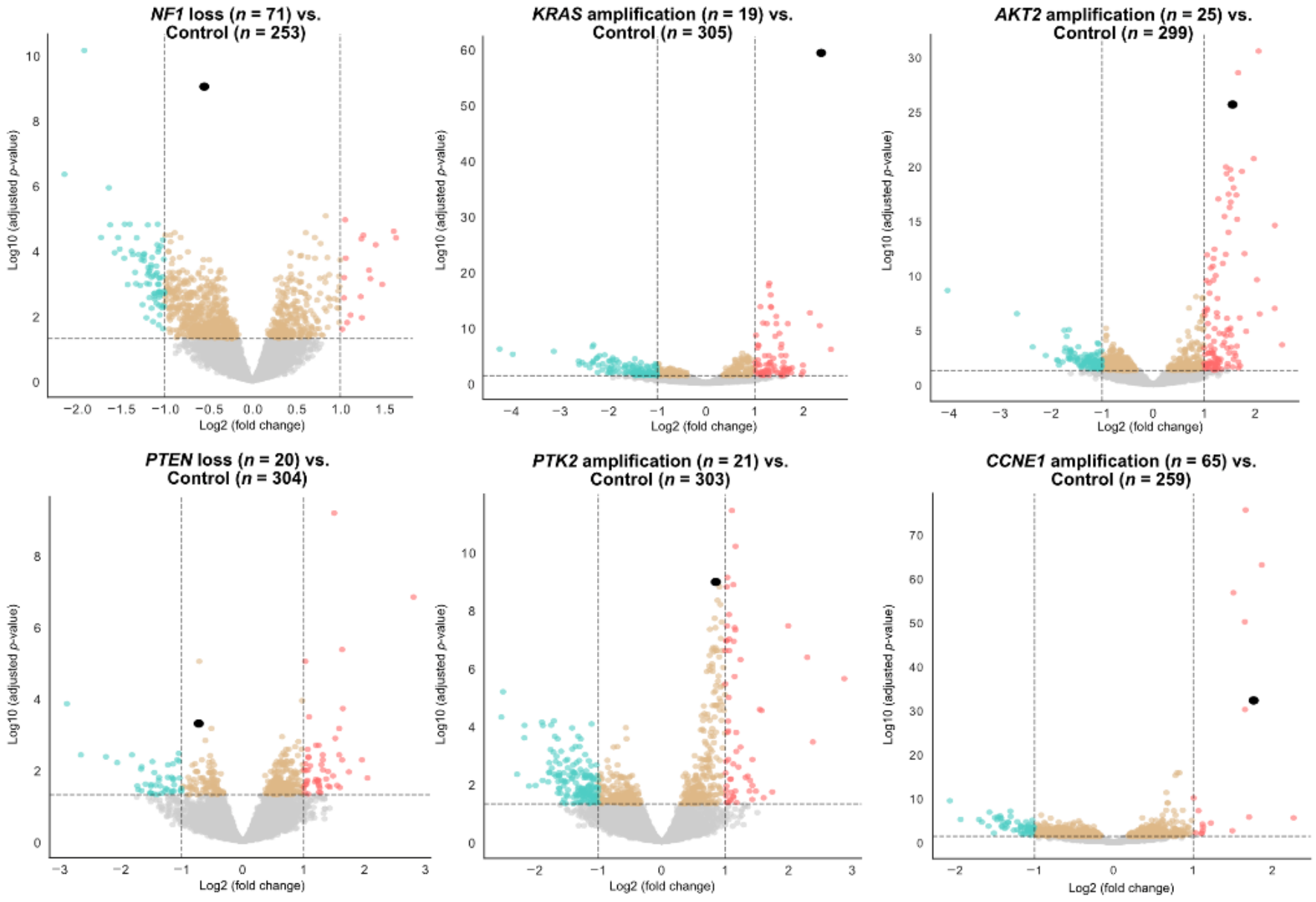

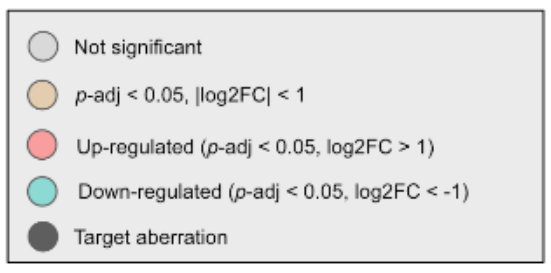

	UP	DOWN	significant
NF1 loss	17	77	1,001
KRAS amp	81	114	452
AKT2 amp	117	96	780
PTEN loss	51	45	394
PTK2 amp	60	160	682
CCNE1 amp	18	53	1,149

Table S1. Clinical characteristics of the 335 study patients.

	Category	*n*	%
**Diagnosis year**	2009-2014	40	11.9 %
	2015-2019	141	42.1 %
	2020-2024	154	46.0 %
**Age at diagnosis**	35-59	47	14.0 %
	60-69	117	34.9 %
	70-79	145	43.3 %
	80-89	26	7.8 %
**Stage (FIGO 20141)** [42]	IA-IIIB	34	10.1 %
	IIIC	203	60.6 %
	IVA-B	98	29.3 %
**Treatment strategy**	PDS	183	54.6 %
	NACT	152	45.4 %
**Outcome from primary therapy (RECIST 1.12)** [43]	Complete Response	175	52.2 %
	Partial Response	108	32.2 %
	Stable Disease	12	3.6 %
	Progressive Disease	23	6.9 %
	ND	17	5.1 %
**PFI (IQR)**	8.6 months (3.9 months - 17.3 months)		
**5-year OS (95% CI)**	33.7% (28.2% - 40.4%)		

PDS: Primary debulking surgery, NACT: Neoadjuvant chemotherapy, PFI: Platinum-free interval, IQR: Interquartile range, OS: Overall survival, CI: Confidence interval

Table S3. Drug concentrations

Target	Compound	Concentrations
SHP2	RMC-4550	100, 500 nM
SOS1	BI-3406	100, 500 nM
KRAS	RMC-7977	40, 200 nM
MEK	cobimetinib	100, 500 nM
ERK	MK-8353	100, 500 nM
-	carboplatin	Sample-specific
PARP	saruparib	50, 250 nM
BCL-2/BCL-XL	navitoclax	100, 500 nM
CDK4/6	palbociclib	100, 500 nM
PKMYT-1	RP6306	50, 250 nM
WEE1	adavosertib	30, 150 nM
ATR	ceralasertib	200, 1000 nM
CHEK1	prexasertib	10, 50 nM

References

1. Lahtinen A, Lavikka K, Virtanen A, Li Y, Jamalzadeh S, Skorda A, et al. Evolutionary states and trajectories characterized by distinct pathways stratify patients with ovarian high grade serous carcinoma. Cancer Cell. 2023;41:1103-1117.e12. https://doi.org/10.1016/j.ccell.2023.04.017

2. Micoli G, Lavikka K, Li Y, Pirttikoski A, Afenteva D, Senkowski W, et al. Decoding the Genomic and Functional Landscape of Emerging Subtypes in Ovarian Cancer. Cancer Discov. 2025;15:2262–77. https://doi.org/10.1158/2159-8290.CD-25-0652

3. Afenteva D, Yu R, Rajavuori A, Salvadores M, Launonen IM, Lavikka K, et al. Multi-omics analysis reveals the attenuation of the interferon pathway as a driver of chemo-refractory ovarian cancer. Cell Reports Med. 2025;6. https://doi.org/10.1016/j.xcrm.2025.102316

4. Cervera A, Rantanen V, Ovaska K, Laakso M, Nuñez-Fontarnau J, Alkodsi A, et al. Anduril 2: Upgraded large-scale data integration framework. Bioinformatics. 2019;35. https://doi.org/10.1093/bioinformatics/btz133

5. Bolger AM, Lohse M, Usadel B. Trimmomatic: a flexible trimmer for Illumina sequence data. Bioinformatics. 2014;30:2114–20. https://doi.org/10.1093/bioinformatics/btu170

6. Li H. Aligning sequence reads, clone sequences and assembly contigs with BWA-MEM. arXiv. 2013.

7. Picard Toolkit [Internet]. Broad Institute, GitHub Repos. Broad Institute; 2019 [cited 2026 Jun 18]. https://broadinstitute.github.io/picard/. Accessed 18 Jun 2026

8. Van der Auwera GA, O'Connor BD. Genomics in the Cloud: Using Docker, GATK, and WDL in Terra. 1st Edition. Sebastopol, CA: O'Reilly Media; 2020.

9. Zhang F, Flickinger M, Taliun SAG, Abecasis GR, Scott LJ, McCaroll SA, et al. Ancestry-agnostic estimation of DNA sample contamination from sequence reads. Genome Res. 2020;30:185–94. https://doi.org/10.1101/gr.246934.118

10. Wang K, Li M, Hakonarson H. ANNOVAR: functional annotation of genetic variants from high-throughput sequencing data. Nucleic Acids Res. 2010;38:e164–e164. https://doi.org/10.1093/nar/gkq603

11. Morales J, Pujar S, Loveland JE, Astashyn A, Bennett R, Berry A, et al. A joint NCBI and EMBL-EBI transcript set for clinical genomics and research. Nature. 2022;604:310–5. https://doi.org/10.1038/s41586-022-04558-8

12. Tamborero D, Rubio-Perez C, Deu-Pons J, Schroeder MP, Vivancos A, Rovira A, et al. Cancer Genome Interpreter annotates the biological and clinical relevance of tumor alterations. Genome Med. 2018;10:25. https://doi.org/10.1186/s13073-018-0531-8

13. Cheng J, Novati G, Pan J, Bycroft C, Žemgulyte A, Applebaum T, et al. Accurate proteome-wide missense variant effect prediction with AlphaMissense. Science (80-). 2023;381. https://doi.org/10.1126/science.adg7492

14. Adzhubei I, Jordan DM, Sunyaev SR. Predicting functional effect of human missense

mutations using PolyPhen-2. Curr Protoc Hum Genet. 2013; https://doi.org/10.1002/0471142905.hg0720s76

15. Vaser R, Adusumalli S, Leng SN, Sikic M, Ng PC. SIFT missense predictions for genomes. Nat Protoc. 2016;11:1–9. https://doi.org/10.1038/nprot.2015.123

16. Landrum MJ, Chitipiralla S, Kaur K, Brown G, Chen C, Hart J, et al. ClinVar: updates to support classifications of both germline and somatic variants. Nucleic Acids Res. 2025;53:D1313–21. https://doi.org/10.1093/nar/gkae1090

17. Chakravarty D, Gao J, Phillips S, Kundra R, Zhang H, Wang J, et al. OncoKB: A Precision Oncology Knowledge Base. JCO Precis Oncol. 2017; https://doi.org/10.1200/po.17.00011

18. Jian X, Boerwinkle E, Liu X. *In silico* prediction of splice-altering single nucleotide variants in the human genome. Nucleic Acids Res. 2014;42:13534–44. https://doi.org/10.1093/nar/gku1206

19. Perez-Villatoro F, Oikkonen J, Casado J, Chernenko A, Gulhan DC, Tumiati M, et al. Optimized detection of homologous recombination deficiency improves the prediction of clinical outcomes in cancer. npj Precis Oncol. 2022;6:96. https://doi.org/10.1038/s41698-022-00339-8

20. Depristo MA, Banks E, Poplin R, Garimella K V., Maguire JR, Hartl C, et al. A framework for variation discovery and genotyping using next-generation DNA sequencing data. Nat Genet. 2011;43. https://doi.org/10.1038/ng.806

21. Van Loo P, Nordgard SH, Lingjærde OC, Russnes HG, Rye IH, Sun W, et al. Allele-specific copy number analysis of tumors. Proc Natl Acad Sci. 2010;107:16910–5. https://doi.org/10.1073/pnas.1009843107

22. Lavikka K, Oikkonen J, Li Y, Muranen T, Micoli G, Marchi G, et al. Deciphering cancer genomes with GenomeSpy: a grammar-based visualization toolkit. Gigascience. 2024;13. https://doi.org/10.1093/gigascience/giae040

23. Cameron DL, Schröder J, Penington JS, Do H, Molania R, Dobrovic A, et al. GRIDSS: sensitive and specific genomic rearrangement detection using positional de Bruijn graph assembly. Genome Res. 2017;27:2050–60. https://doi.org/10.1101/gr.222109.117

24. Priestley P, Baber J, Lolkema MP, Steeghs N, de Bruijn E, Shale C, et al. Pan-cancer whole-genome analyses of metastatic solid tumours. Nature. 2019;575:210–6. https://doi.org/10.1038/s41586-019-1689-y

25. R Core Team. R: A language and Environment for Statistical Computing. Vienna, Austria: R Foundation for Statistical Computing; 2022.

26. Lavikka K, Maarala AI, Oikkonen J, Hautaniemi S. Jellyfish: integrative visualization of spatio-temporal tumor evolution and clonal dynamics. Bioinformatics. 2025;41. https://doi.org/10.1093/bioinformatics/btaf091

27. Icay K, Chen P, Cervera A, Rantanen V, Lehtonen R, Hautaniemi S. SePIA: RNA and small RNA sequence processing, integration, and analysis. BioData Min. 2016;9:20. https://doi.org/10.1186/s13040-016-0099-z

28. Dobin A, Davis CA, Schlesinger F, Drenkow J, Zaleski C, Jha S, et al. STAR: ultrafast

universal RNA-seq aligner. Bioinformatics. 2013;29:15–21. https://doi.org/10.1093/bioinformatics/bts635

29. Roberts A, Pachter L. Streaming fragment assignment for real-time analysis of sequencing experiments. Nat Methods. 2013;10:71–3. https://doi.org/10.1038/nmeth.2251

30. Häkkinen A, Zhang K, Alkodsi A, Andersson N, Erkan EP, Dai J, et al. PRISM: recovering cell-type-specific expression profiles from individual composite RNA-seq samples. Bioinformatics. 2021;37:2882–8. https://doi.org/10.1093/bioinformatics/btab178

31. Love MI, Huber W, Anders S. Moderated estimation of fold change and dispersion for RNA-seq data with DESeq2. Genome Biol. 2014;15:550. https://doi.org/10.1186/s13059-014-0550-8

32. Ewels PA, Peltzer A, Fillinger S, Patel H, Alneberg J, Wilm A, et al. The nf-core framework for community-curated bioinformatics pipelines. Nat Biotechnol. 2020;38:276–8. https://doi.org/10.1038/s41587-020-0439-x

33. Uhrig S, Ellermann J, Walther T, Burkhardt P, Fröhlich M, Hutter B, et al. Accurate and efficient detection of gene fusions from RNA sequencing data. Genome Res. 2021;31:448–60. https://doi.org/10.1101/gr.257246.119

34. Haas BJ, Dobin A, Li B, Stransky N, Pochet N, Regev A. Accuracy assessment of fusion transcript detection via read-mapping and de novo fusion transcript assembly-based methods. Genome Biol. 2019;20:213. https://doi.org/10.1186/s13059-019-1842-9

35. Melsted P, Hateley S, Joseph IC, Pimentel H, Bray N, Pachter L. Fusion detection and quantification by pseudoalignment. 2017. https://doi.org/10.1101/166322

36. Ma C, Shao M, Kingsford C. SQUID: transcriptomic structural variation detection from RNA-seq. Genome Biol. 2018;19:52. https://doi.org/10.1186/s13059-018-1421-5

37. Schubert M, Klinger B, Klünemann M, Sieber A, Uhlitz F, Sauer S, et al. Perturbation-response genes reveal signaling footprints in cancer gene expression. Nat Commun. 2018;9:20. https://doi.org/10.1038/s41467-017-02391-6

38. Müller-Dott S, Tsirvouli E, Vazquez M, Ramirez Flores RO, Badia-i-Mompel P, Fallegger R, et al. Expanding the coverage of regulons from high-confidence prior knowledge for accurate estimation of transcription factor activities. Nucleic Acids Res. 2023;51:10934–49. https://doi.org/10.1093/nar/gkad841

39. Badia-i-Mompel P, Vélez Santiago J, Braunger J, Geiss C, Dimitrov D, Müller-Dott S, et al. decoupleR: ensemble of computational methods to infer biological activities from omics data. Bioinforma Adv. 2022;2. https://doi.org/10.1093/bioadv/vbac016

40. Muzellec B, Teleńczuk M, Cabeli V, Andreux M. PyDESeq2: a python package for bulk RNA-seq differential expression analysis. Bioinformatics. 2023;39. https://doi.org/10.1093/bioinformatics/btad547

41. Buniello A, Suveges D, Cruz-Castillo C, Llinares MB, Cornu H, Lopez I, et al. Open Targets Platform: Facilitating therapeutic hypotheses building in drug discovery. Nucleic Acids Res. 2025;53. https://doi.org/10.1093/nar/gkae1128

42. Prat J. Staging classification for cancer of the ovary, fallopian tube, and peritoneum. Int J

Gynecol Obstet. 2014;124:1–5. https://doi.org/10.1016/j.ijgo.2013.10.001

43. Eisenhauer EA, Therasse P, Bogaerts J, Schwartz LH, Sargent D, Ford R, et al. New response evaluation criteria in solid tumours: Revised RECIST guideline (version 1.1). Eur J Cancer. 2009;45:228–47. https://doi.org/10.1016/j.ejca.2008.10.026
